# Effect of *Salvia miltiorrhiza* aerial parts on growth performance, nutrient digestibility, and digestive enzymes in rabbits

**DOI:** 10.5713/ab.21.0070

**Published:** 2021-06-23

**Authors:** Jiajia Wang, Yiran Luo, Pei Li, Feike Zhang, Ning Liu

**Affiliations:** 1 Department of Animal Science, Henan University of Science and Technology, Luoyang 471023, China; 2 Luoyang Xintai Agro-pastoral Technology Co., Ltd, Luoyang 471400, China

**Keywords:** Digestive Enzyme, Growth Performance, Nutrient Digestibility, Rabbit, *Salvia miltiorrhiza* Aerial Parts

## Abstract

**Objective:**

This study aimed to investigate the effect of *Salvia miltiorrhiza* (*S.m*.) aerial parts as an alternative ingredient on growth performance, nutrient digestibility, and digestive enzymes in growing rabbits.

**Methods:**

Treatments included five tested diets: a control (basal diet), antibiotic (basal diet +enramycin at 5 mg/kg), and *S.m*. aerial parts powder added at 3.0%, 6.0%, and 9.0% of feed using 300 growing rabbits.

**Results:**

The diets with *S.m*. aerial parts addition at 9.0% decreased (p<0.05) feed/gain compared to the control, but there were no differences in feed intake and body weight gain. In contrast with the control, the addition of antibiotic increased (p<0.05) digestibility of dry matter, crude protein, energy, fiber, and ash. The herb addition did not cause differences in the digestibility of most nutrients compared to the antibiotic, but fiber digestibility of the herb at 6.0% and 9.0% was lower (p<0.05) than that of the antibiotic. Moreover, the antibiotic and the herb also similarly increased (p<0.05) the activities of duodenal α-amylase, maltase, lipase, and trypsin, compared to the control, and the herb at 6.0% and 9.0% showed a greater (p<0.05) activity of elastase than the dose 3.0%.

**Conclusion:**

The obtained data indicate that *S.m*. aerial parts can be a potential forage in rabbit’s diet at 9.0% with a beneficial regulation on nutrition and digestion.

## INTRODUCTION

*Salvia miltiorrhiza* (*S.m*.) is a perennial herbaceous medicinal plant 30 to 80 cm in height. Recently, clinical studies have confirmed that *S.m*. crude extract or active components had the ability to treat diabetic, cardiovascular, and neurodegenerative diseases [1–3]. The action mechanism of *S.m*. relies on the amelioration of oxidative stress by regulating antioxidant systems, such as superoxide dismutase, catalase, and glutathione [4]. The popularity of *S.m*. phytochemicals lead to a strong market demand and consequent large artificial planted area for obtaining its root while aerial parts including stems, leaves, and flowers are also worthy of investigation.

The significant pharmacological components extracted from *S.m*. can be mainly divided into two categories: fat-soluble tanshinone compounds and water-soluble salvianolic acids which are representative active ingredients in herbal medicine [5,6]. Given the function against oxidation, inflammation, allergy, and cancer and regulation of tissue repair and regeneration, whether the aerial parts of *S.m*. can be a forage with a growth-promoting effect in farm animals is unclear. Moreover, no literature reports the relationship between *S.m*., nutrient digestibility, and digestive enzyme activity.

Therefore, for the exploration of new forage resources and maximum utilization of by-products from the human consumption, the present study aimed to investigate the effect of *S.m*. aerial parts as an alternative ingredient on growth performance, nutrient digestibility, and activities of digestive enzymes in farm rabbits compared to controls with/without a growth-promoting antibiotic.

## MATERIALS AND METHODS

### Animal care

The experimental protocol of the present study was approved by the Institutional Committee for Animal Use and Ethics of Henan University of Science and Technology (No. 2018010).

### *Salvia miltiorrhiza* and diets

The *S.m*. was planted in Funiu mountains in Luoyang of China (112°17′ N, 34°52′ E) and aerial parts were harvested during the last week of flowering, air dried, ground into meal (40-mesh sieve) and added at 3.0%, 6.0%, and 9.0% in diets [7]. The chemical composition of aerial parts of *S.m*. was listed in Table 1.

The nutrition levels of diets and animal management were referred to Technical Specification for Feeding and Management of Hyla Rabbits (Table 2). All diets were considered as isoenergetic and isonitrogenic and were fed as cold formed pellets (diameter×length, 3.5×8.0 mm) with water content under 14% [8].

### Animals and samples

A total of 300 weaned male Hyla rabbits at approximately 35 days old with initial body weight 1,062 g±17.8 (mean±standard deviation) were randomly assigned to 5 groups with 6 replicates of 10 rabbits each. Rabbits per replicate were housed individually in a continuous row cage [9,10] and all replicates were uniformly distributed in the rabbit house to minimize spatial errors. The rabbits had free access to the diet and water and free of medication other than experimental factors. The feeding trial lasted for 28 d after an adjustment period of 5 d. Rabbits and feed in each replicate were weighed weekly. Average daily feed intake (ADFI), average daily body weight gain (ADG), and feed/gain were immediately adjusted when mortality occurred. All rabbits were monitored for general health twice a day throughout the trial.

At 26 to 28 d of the feeding trial, fresh feces from individual rabbits were collected, pooled by replicate, and prepared for the analysis of nutrient digestibility [11]. Care was taken during the collection of fecal samples to avoid contamination from fur and other foreign materials. At 28 d, 5 rabbits per replicate were randomly selected, suffocated by carbon dioxide, and then the duodenum were dissected and cleaned with phosphate-buffered saline (0°C to 4°C) and mucosa samples were collected and stored at −40°C for digestive enzyme assay [12].

### Chemical and biochemical analysis

Chemical analysis of proximate nutrients in diets and *S.m*. aerial parts was carried out according to the method by Liu et al [11]. Total tanshinone compounds and phenolic acids in *S.m*. were detected according to Pharmacopoeia of China (Volume I, 2015) by high performance liquid chromatography and thin layer chromatography, respectively.

Assay kits from Nanjing Jiancheng Biological Institute (Nanjing, China) were used for the detection of lipase (A054-1-1), α-amylase (C016-2-1), sucrase (A082-2-1), maltase (A082-3-1), trypsin (A080-2-2), and elastase (H198). All samples were carried out in triplicate according to the specifications of kits. The units of enzymes were finally calculated and expressed as per mg of protein in the supernatant of tissue samples to minimize the errors during sample preparation.

### Statistics

Data were subjected to analysis of variance and means (n = 6) were separated by Tukey’s-b test at p<0.05 using IBM SPSS (version 23). Statistical unit for growth performance and nutrient digestibility was from the average of 10 rabbits per replicate and intestinal digestive enzymes from 5 anatomic rabbits.

## RESULTS

### *Salvia miltiorrhiza* and growth performance

Mortality of rabbits throughout the experiment was <5%, not statistically significant among treatments (data not shown). The diets supplemented with *S.m*. aerial parts at 3.0%, 6.0%, and 9.0% had no significant effects on ADFI and ADG (Table 3), while the dose at 9.0% decreased (p<0.05) feed/gain by 6.2% compared to the control, but there were no differences among other treatments.

### *Salvia miltiorrhiza* and nutrient digestibility

The antibiotic addition increased (p<0.05) the digestibility of dry matter, crude protein, energy, ADF, NDF, and minerals, compared to the control (Table 4). For *S.m*. aerial parts, similar results were found with these nutrients except for ADF and NDF which were lower (p<0.05) than the antibiotic but similar to the control.

### *Salvia miltiorrhiza* and digestive enzymes

Significant treatment effects (p<0.05) were found with the activity of α-amylase, maltase, lipase, trypsin, and elastase, except for sucrose (Table 5). Antibiotic supplementation resulted in a higher (p<0.05) enzyme activity than the control group for α-amylase, maltase, lipase, and trypsin. The herb at the three doses and the antibiotic had similar effects on these enzymes, but there were no significant dose effects. For elastase, the antibiotic, and the herb at 6.0% and 9.0% did not increase its activity, and the dose 3.0% exhibited the lowest (p<0.05) activity.

## DISCUSSION

Root plants including *S.m*. are well-known for their root utilization but more than half of photosynthesis products are deposited in aerial leaves and stems which can be a good forage source for herbivores [13]. Especially under artificial planting, aerial parts of root crops have a high yield [5]. In the present study, mowing of *S.m*. was arranged at the last week of the flowering period that lasts approximate 6 months, thereby leading to less lignification, higher nutritional values reflecting on crude fiber and crude protein and a non-significant negative effect on root growth and quality (data not shown) from the serial pilot studies of authors. The proximate nutrient contents primarily indicate that S.m. aerial parts can be a forage in the rabbit feed.

The *S.m*. aerial parts at the three doses did not cause differences in ADFI and ADG of rabbits among treatments. Literature showed that tanshinone IIA increased ADG of broilers with pulmonary arterial hypertension [14]; *Salvia* root improved egg performance of hens [15]; however, salvianolic acid B prevented ADG in high-fat diet-induced obese mice [16]. These inconsistencies in feed consumption and growth by feeding aerial parts, root, or bioactive compounds of *S.m*. to animals need further studies. Interestingly, the feed efficiency in the herb at 9.0% was greater, demonstrating its beneficially physiological regulation. This may be due to the bioactive components of *S.m*. because tanshinones and salvianolic acids are capable against oxidative stress and inflammation [3,17]. Furthermore, the comparable ADG between the herb and the antibiotic implies the potential growth-promoting effect of *S.m*. aerial parts.

In the present study, in contrast with the control, supplementation of the antibiotic and the herb resulted in significant increases in the digestibility of nutrients of rabbits. The improved feed efficiency and nutrient digestibility may also be attributed to health benefits of tanshinones and salvianolic acids of *S.m*. Indeed, *S.m*. root improved intestinal barrier integrity, physiological metabolism, and amino acids absorption [18,19]. Paradoxically, in a rodent model, tanshinones and salvianolic acids depressed nutrient deposition and modulated gut microbiota [16,20]. Information is however unavailable about the effect of *S.m*. aerial parts on the growth performance and nutrient digestion in farm animals. In the present study, significant increases in nutrient digestibility of diets with the herb addition indicate its beneficial regulation on the digestive physiology of rabbits.

Indeed, the activities of digestive enzymes including α-amylase, sucrase, maltase, lipase, and trypsin were increased in diets containing *S.m*. aerial parts. However, high doses of flavonoids from *Salvia chlorolueca* aerial parts showed inhibitory effects on α-amylase and α-glucosidase [21]. Similarly, cryptotanshinone or tanshinone IIA from *S.m*. at 1% inhibited pancreatic lipase activity [22]. Jordan indigenous *Salvia* spp. extracts depressed gastrointestinal carbohydrate and lipid digestion and absorption [23]. Besides the flavonoid concentration, the underlying reason why the extracted flavonoids and their raw materials showed reverse effects on carbohydrases and lipase deserves further study, and molecular docking studies will be needed to establish whether a direct interaction of the principal constituents of the *S.m*. with digestive enzymes can be evoked. Importantly, the herb also improved protein digestion, but literature is unavailable about *S.m*. or its extracts on the activity of proteases, which needs further study. Additionally, with increasing threats from the environment and pathogens, intestinal antioxidant activity of farm animals becomes especially important [24–27], whether the dietary aerial parts of *S.m*. influence intestinal health is also an interesting topic.

## CONCLUSION

Supplementation of *S.m*. aerial parts at the dose 9.0% decreased feed/gain compared to the control. The antibiotic exhibited significant improvement in the digestibility of dry matter, crude protein, energy, fiber, and ash, and similar results were found for the herb for most nutrients except for fiber which was lower than the antibiotic but similar to the control. The antibiotic and the herb had similar effects on the activities of α-amylase, sucrase, maltase, lipase, and trypsin. The results suggest that *S.m*. aerial parts can be a versatile forage with nutritional and growth-promoting effects on herbivores.

## Figures and Tables

**Table 1:** Chemical compositions of *Salvia miltiorrhiza* aerial parts (% of dry matter)

Composition	Content	SD
Dry matter	87.1	2.296
Crude protein	11.7	0.208
NDF	60.2	2.023
ADF	41.3	1.762
Ca	0.14	0.005
P	0.19	0.005
Ash	6.92	0.174
Tanshinones	0.63	0.021
Salvianolic acids	0.74	0.031

SD, standard deviation; ADF, acid detergent fiber; NDF neutral detergent fiber.

**Table 2:** Ingredients and nutrient levels of basal diet and diets containing *Salvia miltiorrhiza* aerial parts

Item	Basal diet	Aerial parts		
Ingredient (% as fed)				
Aerial parts	0	3.0	6.0	9.0
Alfalfa meal	35.0	32.0	31.0	31.0
Corn	25.0	25.0	25.0	25.0
Soybean meal	10.0	10.0	10.0	11.0
Brewers dried grain	15.0	15.0	13.0	11.0
Wheat bran	10.0	10.0	10.0	8.0
Premix^1)^	5.0	5.0	5.0	5.0
Nutrients^2)^ (% of dry matter)				
Crude protein	16.5	16.4	16.1	16.2
Digestible energy (MJ/kg)	10.53	10.52	10.46	10.37
Acid detergent fiber	15.7	16.1	16.3	16.8
Neutral detergent fiber	23.4	24.0	24.2	24.7
Ash	6.61	6.57	6.63	6.58

1) The premix provided the following per kg of the diet: vit A, 6,000 IU; vit D, 1,000 IU; vit E, 38 mg; Cu, 9 mg; Fe, 50 mg; Mn, 9.0 mg; Zn, 40 mg; dicalcium phosphate, 25 g; NaCl, 4.0 g; Lys, 2.4 g; Met, 1.6 g.

2) Digestible energy was calculated by Chinese Feed Database (version 28, 2017) and others were measured.

**Table 3:** Effect of *Salvia miltiorrhiza* aerial parts on the growth performance of rabbits

Item	Control	Antibiotic	Supplemental aerial parts			SEM	p-value

			3.0%	6.0%	9.0%		
Initial BW (kg/rabbit)	1.07	1.06	1.07	1.05	1.06	0.049	0.998
Final BW (kg/rabbit)	2.65	2.74	2.66	2.68	2.60	0.057	0.188
ADFI (g/rabbit/d)	164	167	159	160	150	7.786	0.283
ADG (g/rabbit/d)	56.4	60.3	56.8	58.2	55.3	1.633	0.056
Feed/gain (g/g)	2.91^a^	2.77^ab^	2.80^ab^	2.75^ab^	2.73^b^	0.082	0.049

SEM, standard error of mean; BW, body weight; ADFI, average daily feed intake; ADG, average daily BW gain.

a,b Means in a row without the same superscript are different, p<0.05.

**Table 4:** Effect of *Salvia miltiorrhiza* aerial parts on the nutrient digestibility of rabbits

Item	Control	Antibiotic	Supplemental aerial parts			SEM	p-value

			3.0%	6.0%	9.0%		
Dry matter (%)	55.5^b^	61.3^a^	55.8^b^	58.2^ab^	60.8^a^	0.731	0.048
Gross energy (%)	54.9^b^	61.5^a^	58.1^ab^	61.2^a^	61.6^a^	0.714	0.043
Crude protein (%)	74.2^b^	82.3^a^	78.5^ab^	79.9^a^	82.2^a^	1.216	0.046
NDF (%)	39.5^b^	48.8^a^	44.0^ab^	39.7^b^	41.5^b^	1.429	0.012
ADF (%)	13.9^b^	25.2^a^	22.6^a^	15.8^b^	17.3^b^	2.253	0.005
Ash (%)	38.5^c^	55.6^a^	53.4^ab^	52.6^b^	59.8^a^	2.226	0.008

SEM, standard error of mean; NDF neutral detergent fiber; ADF, acid detergent fiber.

a–c Means in a row without the same superscript are different, p<0.05.

**Table 5:** Effect of *Salvia miltiorrhiza* aerial parts on the duodenal digestive enzymes of rabbits

Item	Control	Antibiotic	Supplemental aerial parts			SEM	p-value

			3.0%	6.0%	9.0%		
α-Amylase (U/mg)	14.0^b^	19.8^a^	17.2^ab^	20.1^a^	19.9^a^	0.535	0.038
Sucrase (U/mg)	1.68	1.57	1.58	1.69	1.61	0.058	0.872
Maltase (U/mg)	2.15^b^	2.47^a^	2.34^ab^	2.39^ab^	2.52^a^	0.066	0.046
Lipase (U/g)	1.59^b^	1.72^a^	1.65^ab^	1.70^a^	1.73^a^	0.034	0.048
Trypsin (U/mg)	3.17^b^	3.59^a^	3.21^ab^	3.53^a^	3.65^a^	0.074	0.046
Elastase (U/mg)	0.25^a^	0.28^a^	0.20^b^	0.27^a^	0.28^a^	0.011	0.032

SEM, standard error of mean.

a,b Means in a row without the same superscript are different, p<0.05.
